# White matter injury, cholesterol dysmetabolism, and APP/Abeta dysmetabolism interact to produce Alzheimer’s disease (AD) neuropathology: A hypothesis and review

**DOI:** 10.3389/fnagi.2023.1096206

**Published:** 2023-02-10

**Authors:** Frank R. Sharp, Charles S. DeCarli, Lee-Way Jin, Xinhua Zhan

**Affiliations:** Department of Neurology, The MIND Institute, University of California at Davis Medical Center, Sacramento, CA, United States

**Keywords:** white matter, myelin, cholesterol, APOE, Abeta, Alzheimer’s disease, amyloid, tau

## Abstract

We postulate that myelin injury contributes to cholesterol release from myelin and cholesterol dysmetabolism which contributes to Abeta dysmetabolism, and combined with genetic and AD risk factors, leads to increased Abeta and amyloid plaques. Increased Abeta damages myelin to form a vicious injury cycle. Thus, white matter injury, cholesterol dysmetabolism and Abeta dysmetabolism interact to produce or worsen AD neuropathology. The amyloid cascade is the leading hypothesis for the cause of Alzheimer’s disease (AD). The failure of clinical trials based on this hypothesis has raised other possibilities. Even with a possible new success (Lecanemab), it is not clear whether this is a cause or a result of the disease. With the discovery in 1993 that the apolipoprotein E type 4 allele (APOE4) was the major risk factor for sporadic, late-onset AD (LOAD), there has been increasing interest in cholesterol in AD since APOE is a major cholesterol transporter. Recent studies show that cholesterol metabolism is intricately involved with Abeta (Aβ)/amyloid transport and metabolism, with cholesterol down-regulating the Aβ LRP1 transporter and upregulating the Aβ RAGE receptor, both of which would increase brain Aβ. Moreover, manipulating cholesterol transport and metabolism in rodent AD models can ameliorate pathology and cognitive deficits, or worsen them depending upon the manipulation. Though white matter (WM) injury has been noted in AD brain since Alzheimer’s initial observations, recent studies have shown abnormal white matter in every AD brain. Moreover, there is age-related WM injury in normal individuals that occurs earlier and is worse with the APOE4 genotype. Moreover, WM injury precedes formation of plaques and tangles in human Familial Alzheimer’s disease (FAD) and precedes plaque formation in rodent AD models. Restoring WM in rodent AD models improves cognition without affecting AD pathology. Thus, we postulate that the amyloid cascade, cholesterol dysmetabolism and white matter injury interact to produce and/or worsen AD pathology. We further postulate that the primary initiating event could be related to any of the three, with age a major factor for WM injury, diet and APOE4 and other genes a factor for cholesterol dysmetabolism, and FAD and other genes for Abeta dysmetabolism.

## Introduction

Currently, most would consider the amyloid/Abeta cascade hypothesis to be the leading candidate for what causes Alzheimer’s disease (AD) (Selkoe, 2011; Selkoe and Hardy, 2016). Indeed, this hypothesis has fueled most of the recent clinical trials aimed at removing Abeta/amyloid from the brain. With the failure of almost all of these trials (Asher and Priefer, 2022), there have been a few clinical trials aimed at trying to remove Tau which have also failed (Asher and Priefer, 2022). Thus, questions have arisen as to whether deposition of amyloid and/or Tau is the cause or is a consequence of the disease (Herrup, 2015). This has led to some alternative hypotheses that cholesterol metabolism or other pathways might be involved (Høilund-Carlsen et al., 2022; Rudge, 2022).

With the discovery that Apolipoprotein E ε4 (ApoE4) is the major susceptibility gene for late onset AD (LOAD), and since APOE is the major cholesterol transporter in brain and the body, there has been increasing interest in cholesterol metabolism in AD. Indeed, studies in the last 20 years have shown that cholesterol metabolism is intricately intertwined with Abeta/amyloid transport and metabolism (Dietschy and Turley, 2001; Mulder et al., 2001; Burns M. P. et al., 2003; Papassotiropoulos et al., 2003; Yanagisawa, 2003; Rahman et al., 2005; Michikawa, 2006; Kim et al., 2007; Liu et al., 2007; Fan et al., 2009; Martins et al., 2009; Zhou et al., 2009; Wollmer, 2010; Poirier et al., 2014; Chai et al., 2021; Rhea and Banks, 2021; Wang H. et al., 2021). Indeed, manipulations of cholesterol transport and metabolism can completely ameliorate or worsen AD pathology in mouse AD models, raising the question of whether cholesterol metabolism might be upstream and be a driver or at least a contributor to AD pathology in some AD subjects (Martins et al., 2009; Staurenghi et al., 2021; Rudajev and Novotny, 2022).

Though white matter injury was described by Alzheimer in his first reports of AD brain neuropathology, there has been the general belief that the white matter injury was a consequence of gray matter disease/neuronal cell death. However, interest in white matter injury in AD brain has increased with the realization mostly from MRI studies that white matter injury precedes amyloid plaques and neurofibrillary tangles in human early onset familial AD (FAD), in human late onset AD (LOAD), and in rodent AD models (Sexton et al., 2011; Li et al., 2012; Sharma et al., 2022) (see below). Moreover, high concentrations of Abeta have been shown to damage oligodendrocytes and oligodendrocyte precursor cells (OPCs) as well as myelin itself (see below). In spite of the tremendous increase in knowledge noted above, there has not been a model proposed that provides a connection between Abeta/amyloid transport and metabolism, cholesterol transport and metabolism, and white matter injury. Our purpose is to provide a plausible model and make a connection between all three.

**FIGURE 1:**
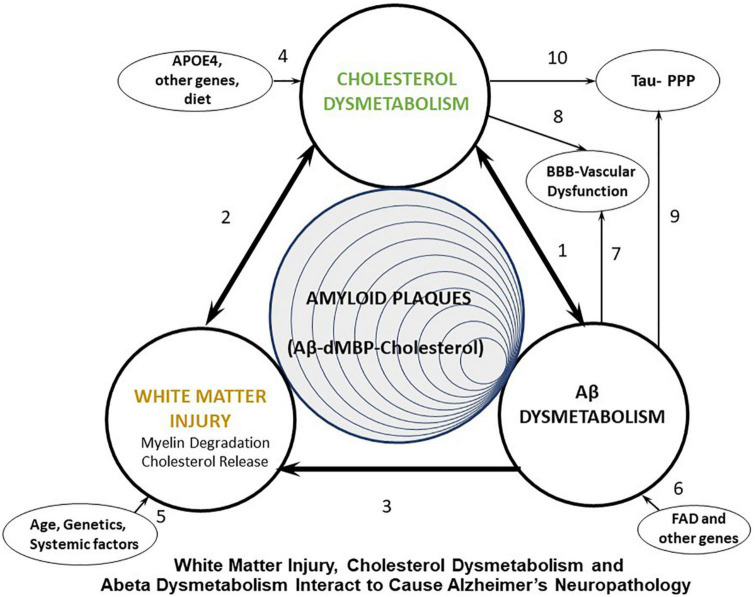
Diagram showing proposed interactions between white matter injury, cholesterol dysmetabolism, and Abeta dysmetabolism that leads to Alzheimer’s plaques, Tau hyperphosphorylation (Tau-PPP), and vascular dysfunction in Alzheimer’s disease (AD). FAD, Familial Alzheimer’s disease (early onset AD); dMBP, degraded myelin basic protein.

**FIGURE 2:**
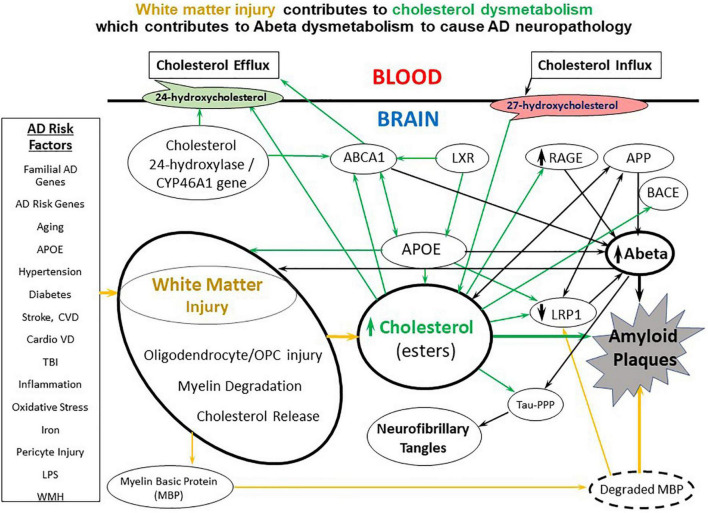
A diagram of the molecular interactions between AD risk factors, white matter injury, cholesterol metabolism and transport, and Abeta metabolism and transport. A variety of environmental, systemic, and genetic factors contribute to white matter injury which causes release of cholesterol from myelin. The white matter cholesterol contributes to cholesterol dysmetabolism along with the APOE4 allele and multiple genetic risk factor genes. Cholesterol has multiple interactions with Abeta including down-regulating LRP1, upregulating RAGE and binding APP to contribute to Abeta dysmetabolism. CVD, cerebrovascular disease; CardioVD, cardiovascular disease; LPS, lipopolysaccharide; WMH, white matter hyperintensities; AD, Alzheimer’s disease; OPC, oligodendrocyte progenitor cells; BBB, blood brain barrier; dMBP, degraded myelin basic protein; APP, amyloid precursor protein; TBI, traumatic brain injury; APOE, apolipoprotein E; LRP1, low density lipoprotein receptor-related protein-1; RAGE, receptor for advanced glycation end products; ABCA1, ATP binding cassette subfamily A member 1; LXR, liver X receptor; BACE, beta secretase 1; Tau-PPP, hyperphosphorylated Tau.

We propose that many systemic factors lead to white matter injury, with increasing age being the number one initiator. Age combined with ApoE status and many other factors, combined with other AD risk factor genes, lead to myelin injury which occurs in white matter and gray matter (Figure 1, arrows 2, 3, 5). The myelin injury leads to cholesterol dysmetabolism in gray and white matter (Figure 1, arrow 2). Increases of cholesterol in brain impair export of Abeta and promote formation of amyloid plaques (Gamba et al., 2015; Figure 1, arrow 1). Cholesterol also binds APP to promote formation of Abeta. Oxidized forms of cholesterol likely promote brain glucose hypometabolism (Gamba et al., 2019; Figure 1, arrow 1). As Abeta levels increase they participate in formation of Abeta aggregates which is promoted by cholesterol and denatured myelin basic protein (MBP), another product of injured myelin. These aggregates form amyloid plaques. Abeta itself is toxic to oligodendrocytes and OPCs and thus also contributes to white matter injury (Figure 1, arrow 3). Thus, a positive feedback injury loop is established with injury to myelin/white matter as the source of cholesterol fuel. Finally, cholesterol and Abeta both contribute to forming hyperphosphorylated tau in neurofibrillary tangles and contribute to vascular injury seen in AD (Figure 1, arrows 7–10). These pathways are shown in greater detail in Figure 2.

We also propose that there are three major groups of AD subjects that are initiated either by White Matter Injury (Age, genetics, systemic factors) (Figure 1, arrow 5), Cholesterol dysmetabolism (APOE4, other genes, diet) (Figure 1, arrow 4) or Abeta dysmetabolism (FAD, other genes) (Figure 1, arrow 6). We now outline the literature supporting these ideas, though this is not an exhaustive review.

## Evidence for white matter injury in AD

### Humans–LOAD

Myelin injury in Alzheimer’s disease (AD) brain was first noted by Alzheimer (Alzheimer et al., 1991; Möller and Graeber, 1998). Following that there were infrequent mentions of white matter pathology in AD brain including those by Terry who found evidence of primary demyelination and lipid-like material passing through the walls of small vessels to enter the lumen in AD brain (Terry et al., 1964). The consensus in the field during the next 60 years, however, was that white matter injury was a consequence of gray matter injury, an idea still held by many (Papuć and Rejdak, 2020). However, it gradually became clear that white matter injury was an important component of AD neuropathology (Englund, 1998), with late myelinating white matter and gray matter layers developing plaques and tangles earlier and in greater numbers than early myelinating areas (retrogenesis theory), with amyloid plaques developing earliest in poorly myelinated gray matter regions (Braak and Braak, 1996; Braak et al., 1999; Stricker et al., 2009; Brickman et al., 2012). Abeta is also deposited in white matter where it is closely associated with blood vessels (Iwamoto et al., 1997).

### MRI studies in LOAD

Interest in white matter (WM) injury was stirred by the development of brain MRI techniques (Diffusion Tensor Imaging–DTI, and Diffusion Imaging) that made it possible to detect early injury to WM (Gold et al., 2012; Radanovic et al., 2013; Nasrabady et al., 2018; Xiao et al., 2022). Bartzokis et al. (2004) examined human brains from normal aging and AD brains during life. They studied the damaged myelin in normal aging and Alzheimer’s disease (AD) brains by evaluating early myelinating and later myelinating regions of the splenium and genu of the corpus callosum. They found changes in myelin beginning at age 31 in normal brain, with later-myelinating regions being more susceptible. This process was worse throughout the AD brain (Bartzokis et al., 2004). Thus, they were among the first to link myelin breakdown beginning in midlife and continuing in the aging brain to the more severe myelin breakdown that they observed in AD brain (Bartzokis et al., 2003). They also showed that the APOE4 allele worsened the white matter injury observed in old healthy individuals (Bartzokis et al., 2007) and were some of the first to emphasize WM injury in AD and its importance to guiding future therapies since the amyloid hypothesis did not at that time help explain WM injury in AD brains (Bartzokis, 2011). As noted below, Abeta can directly injure myelin and oligodendrocytes and could contribute to early WM injury.

A diffusion MRI study of young onset AD showed loss of U fibers (superficial white matter) and dispersion of these fibers in AD (Veale et al., 2021). Moreover, MRI shows greater diffusion restriction in white matter in AD patients (Hanyu et al., 1997) and in preclinical AD where patients are cognitively normal but have positive amyloid positron emission tomography (florbetapir) (Benitez et al., 2022). Other MRI studies show Diffusion Tensor Imaging (DTI) abnormalities in preclinical AD (normal cognition, but abnormal CSF AD markers) (Hoy et al., 2017). The fornix was the most affected WM tract in one AD study (Jin Y. et al., 2017). Another MRI DTI study showed widespread white matter degeneration prior to the onset of dementia (Maier-Hein et al., 2015). A meta-analysis of 227 AD patients and 215 healthy controls with voxel based morphometry showed decreases of white matter volume in AD subjects (Li et al., 2012). Several diffusion indices suggest damage of the white matter is much worse in AD compared to MCI (Mild Cognitive Impairment) (Shu et al., 2011). A meta-analysis including 41 MRI-DTI studies showed abnormal mean diffusivity in most regions of AD and MCI brains (Sexton et al., 2011). Combining 3-dimensional volumetric scans and DTI in AD patients suggests macroscopic white matter atrophy is secondary to gray matter atrophy, while microscopic white matter damage detected by MRI-DTI starts earlier in frontal areas before any macroscopic atrophy in gray and white matter can be detected (Yoon et al., 2011).

A meta-analysis of 1,021 patients in 26 studies showed decreased fractional anisotropy (FA) in AD compared to MCI patients. This occurred in frontal lobe white matter, corpus callosum white matter, fornix and hippocampus, cingulate gyrus and bundle, uncinate and superior longitudinal fasciculus, and inferior fronto-occipital and inferior longitudinal fasciculus (Qin et al., 2021). Thus, though there are changes of WM in MCI, there is a progression of the changes in going from MCI to AD. Not surprisingly, there are alterations in white matter and white matter networks by DTI in preclinical AD (cognitively normal, positive florbetapir-PET or abnormal CSF Abeta) (Molinuevo et al., 2014; Fischer et al., 2015). There are also MRI-DTI abnormalities in white matter of middle aged cognitively normal subjects whose parents had AD (Bendlin et al., 2010).

### Familial early onset AD (FAD)

The Bartzokis group described white matter (WM)changes using MRI-Diffusion Tensor Imaging (DTI) with decreased Fractional Anisotropy (FA) in the WM in preclinical and pre-symptomatic FAD carriers, particularly in late-myelinating tracts connecting limbic areas (Ringman et al., 2007). Decreased FA in the columns of the fornix is particularly robust in early FAD, well before any amyloid plaque and tau pathology (Ringman et al., 2007). Others studies have supported WM injury in FAD (Migliaccio et al., 2012). White matter hyperintensities are a prominent feature of FAD (Schoemaker et al., 2022). Subjects with presenilin FAD mutations have many biochemical alterations of their white matter (Roher et al., 2013). DTI MRI studies have shown WM microstructural damage is more severe in early onset FAD compared to LOAD with the two groups having different topographical distributions of damage (Canu et al., 2013).

### Down syndrome

Most Down’s syndrome patients older than forty develop neuropathology identical to that seen in FAD and LOAD patients. Diffusion Tensor Imaging (DTI-MRI) has shown abnormal myelin in selected white matter tracts in non-demented, young Down syndrome subjects (Romano et al., 2018). Diffusion MRI of Down patients show early changes in late-myelinating and relative sparing of early myelinating pathways, consistent with the “retrogenesis model” proposed for sporadic AD. These late-myelinating tracts correlated with cognitive abnormalities and with regional amyloid deposition observed with Down syndrome (Rosas et al., 2020).

### Neuropathology and molecular studies of white matter injury

There have now been many pathological and molecular studies showing loss of myelin and oligodendrocytes in AD white matter (Brun and Englund, 1986; Sjöbeck et al., 2005; Butt et al., 2019). There are early alterations in oligodendrocytes and oligodendrocyte precursor cells (OPC) and alterations in transcription of myelin-related genes in AD brains that are worse in those with more co-morbidities (Ferrer and Andrés-Benito, 2020). A single cell transcriptomic study of AD brain showed abnormal gene expression in oligodendrocytes and OPCs (Mathys et al., 2019). Transcriptomic network analyses showed abnormalities of two prominent myelin pathways in AD compared to control brains (Humphries et al., 2015).

Cognitive impairment increases with the number of myelin lesions in AD brain which is independent of the amount of amyloid, and appears before any neuronal loss. Myelinating oligodendrocytes in the gray matter are more vulnerable than those in white matter, and the degeneration correlated with the amount oligodendrocyte DNA damage (Tse et al., 2018). Fibrillar Abeta pathology in cortical gray matter occurs in areas of focal demyelination in human presenilin-1 FAD, LOAD, and two mouse AD transgenic models (Mitew et al., 2010). The focal demyelination was greatest in the core of Abeta plaques, with cores showing a focal loss of oligodendrocytes in FAD and LOAD. In human AD and transgenic AD mice that had plaque-free neocortical regions, these showed no evidence of demyelination or loss of oligodendrocytes compared to controls (Mitew et al., 2010).

Lipids comprise 80% of myelin, and the myelin lipids, galactosylceramide, and sulfatide are critical for normal neurological function. One study found marked decreases of galactosylceramide and sulfatide in AD brain which was due to the loss of their biosynthetic precursor, very long chain ceramides (Couttas et al., 2016). Ceramide synthase 2 (CERS2) produces very long chain ceramides. CERS2 activity decreased at an early Braak stage I/II in temporal cortex, and later Braak stage III/IV in hippocampus and frontal cortex, indicating that decreased CERS2 activity precedes formation of cortical neurofibrillary tangles (Couttas et al., 2016). These myelin changes were observed in AD gray matter. Another study found sulfatides depleted as much as 93% in gray matter and 58% in white matter from AD brains of subjects with mild dementia. All other lipid classes except plasmalogen were unaltered. The content of ceramides, a class of potential sulfatide degradation products, was increased three-fold in white matter and peaked with very mild AD dementia (Han et al., 2002). Other studies have noted increased Abeta and decreased cholesterol and myelin proteins in AD WM (Roher et al., 2002) as well as decreased lipids (Wallin et al., 1989). Lipid peroxidation is a prominent feature of AD brain (Chia et al., 1984). APP (amyloid precursor protein) is prominent in AD white matter (Sapirstein et al., 1994; Tokuda et al., 1994). Thus, biochemical studies confirm myelin injury seen in MRI studies of AD.

### AD animal models show white matter injury

The 3 × Tg-AD mouse model shows myelin loss at 6 and 12 months of age. There is a corresponding oligodendrocyte progenitor cell (OPCs) loss with surviving OPCS showing abnormal structure suggesting OPC dysfunction and defective myelin repair (Vanzulli et al., 2020). There is myelin lipid loss around amyloid plaques coupled with APOE deposition and myelin sheath disruption in 5xFAD mice (Kaya et al., 2020). APPxPS1 transgenic mice show reduced fiber tract volumes in corpus callosum and anterior commissure with axon loss and myelin breakdown (Chen et al., 2011; Dong et al., 2018). Triple-transgenic AD (3 × Tg-AD) mice exhibit myelin abnormalities similar to FAD patients; and the PS1 (M146V) mutation predisposes mouse OPCs to Aβ(1-42) -induced alterations in cell differentiation and function that results in an abnormal distribution of myelin basic protein (MBP) (Desai et al., 2011).

The above studies along with many not cited here have led some investigators to ask “Is Alzheimer’s disease a disease of white matter?” (Sachdev et al., 2013; Nasrabady et al., 2018). This review suggests that white matter injury contributes to cholesterol dysmetabolism which then contributes to Abeta dysmetabolism. One of the central questions that remains unanswered from all of these studies is whether the white matter changes that occur in AD are secondary to very early gray matter/axon injury, or whether they are due to “primary effects” on the myelin (Fletcher et al., 2013). We argue that age, AD risk factor genes and systemic factors can selectively injure oligodendrocytes, OPCs and myelin that results in cholesterol dysmetabolism which tips the scales toward Abeta retention and aggregation and amyloid plaque formation.

## Evidence that white matter (WM) injury can precede ad pathology

### Humans

There is evidence in both early onset FAD and sporadic late onset AD (LOAD) that myelin and oligodendrocyte injury precede the development of amyloid plaques and neurofibrillary tangles (Cai and Xiao, 2016; Butt et al., 2019). A study was performed for 162 healthy 2–25 month-old infants with no family history of any neurological or psychiatric disorders for APOE ε4 carrier compared to non-carrier groups (Dean et al., 2014). The APOE ε4 carrier group had several white and gray matter differences not seen in the non-carriers (Dean et al., 2014). APOEε4 carriers have disrupted rates of cognitive and white matter development in childhood (Remer et al., 2020). Alterations in white matter integrity occur in normal middle-aged women at high risk for AD who either have a family history of AD or at least one APOE4 allele (Gold et al., 2010). White matter microstructure is altered as assessed by DTI-MRI in cognitively normal middle-aged APOE-ε4 homozygotes prior to any Abeta/tau pathology (Operto et al., 2018). An AD family history correlated with lower Fractional Anisotropy (FA) on MRI-DTI in brain regions known to be affected by AD. There was no main effect of APOE4 in one study; however, APOE4 carrier patients with a family history of AD and an APOE4 allele had the most abnormal white matter compared to other groups (Bendlin et al., 2010). APOE4 status affects white matter integrity in young to middle age individuals before amyloid plaque or Tau pathology (Goltermann et al., 2021). The decreased fractional anisotropy in DTI-MRI studies of white matter of mild cognitive impairment (MCI) patients were associated with progression to AD (Lo Buono et al., 2020).

Progression of changes of episodic memory can be predicted in cognitively intact, healthy aged individuals by disruption of white matter microstructure by DTI-MRI in the temporal lobe (Lancaster et al., 2016). These findings were observed in individuals with a high AD risk with a family history of AD and an APOE4 allele. This suggests the white matter disruption is related to early neuropathological changes prior to any cognitive changes or formation of plaques and tangles (Lancaster et al., 2016). DNA-damage to oligodendrocytes occurs before formation of plaques and tangles in AD brain (Tse et al., 2018).

The best evidence for myelin injury occurring prior to formation of plaques and tangles comes from those patients with early onset autosomal dominant, familial AD (FAD). This is because the age of onset of formation of amyloid plaques and tangles is fairly consistent. MRI -diffusion tensor imaging (DTI) of FAD mutation carriers shows white matter abnormalities in late-myelinating tracts before formation of plaques and tangles (Ringman et al., 2007). FAD mutation carriers had greater total White Matter Hyperintensity volumes, which increased 6 years before predicted symptom onset (Lee et al., 2016). The parietal and occipital lobes were affected nearly 22 years before estimated onset (Lee et al., 2016). In another FAD study there was a stronger increase of mean diffusivity by DTI-MRI within parietal and frontal white matter in FAD mutation carriers (Araque Caballero et al., 2018), with changes being observed 5–10 years before predicted symptom onset and correlating with low CSF Abeta1-42 and high tau, P-tau and TREM2 (Araque Caballero et al., 2018).

### AD animal models

All rodent AD models studied show evidence of white matter injury prior to formation of plaques and tangles. Diffusion MRI of 3 × Tg-AD mice showed myelin abnormalities throughout brain including fimbria and fornix before development of AD pathology (Falangola et al., 2020). Myelin basic protein (MBP) immunoreactivity in the fimbria was less in 3 × Tg-AD mice compared to controls. Diffusion MRI detected myelin abnormalities in 2-month-old 3 × Tg-AD mice who develop amyloid plaques by 6 months (Falangola et al., 2020). APP/PS1 AD mice show myelin loss and MBP mRNA and impaired oligodendrocyte development in 2–3 month old mice well before the formation of plaques and tangles (Wu et al., 2017; Dong et al., 2018). Myelin deficits in 5XFAD mice start at month of age and worsen with age (Gu et al., 2018). An index of myelin thickness changed in 1-month-old 5XFAD mice in hippocampus and entorhinal cortex compared to controls and spread to other regions in older mice. 5XFAD mice have spatial memory deficits by 1 month and spatial learning deficits by 2 months of age that correlate with abnormalities of myelin (Gu et al., 2018). Myelin abnormalities occur months before amyloid plaque pathology in 3 × Tg-AD mice (Desai et al., 2009, 2010). There is increased BBB permeability in Tg2576 AD in gray and white matter in mice months before any evidence of plaques and tangles (Ujiie et al., 2003).

Thus, there is increasing evidence for white matter (WM) injury prior to formation of plaques and tangles particularly in FAD and in AD mouse models. The evidence for LOAD is less convincing, though there is evidence for MRI-DTI abnormalities in cognitively normal individuals at high risk for AD including those with a family history of AD and/or an APOE4 allele. These findings require an explanation but may help us understand why so many systemic factors appear to contribute to AD risk, since many systemic factors have significant effects on brain white matter than can accumulate with aging (Figure 2). Age is the number one risk factor for LOAD and since there are increasing white matter abnormalities with aging, this may help explain why aging plays such a prominent role both in FAD and LOAD. These observations do not rule out an important role for Abeta/APP since Abeta has been shown to damage white matter (see next section). Thus, elevations of Abeta prior to formation of plaques might contribute to WM injury (see below).

By linking white matter injury to cholesterol and Abeta metabolism and transport, we postulate that this helps explain why so many systemic factors increase the risk of AD. We postulate that the many AD risk factors act on white matter particularly with aging when the BBB is leakier (Methia et al., 2001; Ujiie et al., 2003; Zipser et al., 2007; Zlokovic, 2008; Deane et al., 2009; Farrall and Wardlaw, 2009; Jaeger et al., 2009; Lamartinière et al., 2018; Ma et al., 2018; Barisano et al., 2022; Wang et al., 2022c). Indeed, BBB dysfunction precedes amyloid plaque formation (Ujiie et al., 2003). Thus, we propose that many AD risk factors contribute to white matter injury and cholesterol dysmetabolism as shown in Figure 2. We now discuss some of the AD risk factors that contribute to WM injury in normal aging and in AD brain.

## Multiple mechanisms of white matter (WM) injury in AD

### Age

Age is the most important factor that determines risk and time of onset of LOAD and early onset/familial AD (Braak and Braak, 1997; Liu et al., 2017). How age predisposes to AD, however, has not been clear (Stahon et al., 2016). One possibility is that age-induced injury to white matter could be the primary reason (Liu et al., 2017; Chen et al., 2020; Sorond et al. (2020)). Indeed, age and APOE-ε4 allele status affect myelin content in white matter of cognitively normal adults (Operto et al., 2019). However, to complicate this assertion it has been found that increasing age decreases LRP1 at the BBB which would decrease Abeta efflux from cells and the BBB, and increasing age increases RAGE at the BBB which would increase Abeta influx into brain (Osgood et al., 2017). Though this would certainly favor formation of amyloid plaques, the increased Abeta with age could also contribute to myelin/white matter injury as noted below. There is also an age-related decrease in oligodendrocyte precursor cells and formation of new oligodendrocytes which decreases white matter injury repair (Miyamoto et al., 2013; Dimovasili et al., 2022). There is also impairment of OPCs developing into mature oligodendrocytes in aging white matter (Bagi et al., 2018). White matter remains vulnerable to normal aging processes through the tenth decade of life (Bennett et al., 2017). There is less cholesterol in aging brain likely as the result of myelin loss (Stommel et al., 1989). A diffusion MRI study showed aging affected white matter microstructure and macrostructure (Schilling et al., 2022). Overall, there is a loss of myelin and oligodendrocytes that progresses with normal aging and in AD brain in spite of an increase of oligodendrocyte precursor cells (Hampton et al., 2012; Chen et al., 2021).

Aging is also associated with increases of cytokines, chemokines, lipopolysaccharide (Hakoupian et al., 2021), inflammation and increased oxidative stress that contribute to white matter injury (Altendahl et al., 2020). Indeed, T cell infiltration of white matter is associated with cognitive decline as normal monkeys age (Batterman et al., 2021). Microglia in white matter associate with myelin injury in both normal aging and in AD brain and are presumed to clear damaged myelin (Safaiyan et al., 2021). With aging NMDA receptors localize to myelin as well as oligodendrocytes, making them more vulnerable to glutamate (Stys and Lipton, 2007; Baltan, 2016). With aging there is a decrease in LRP1 and an increase of RAGE at the BBB which would elevate Abeta levels and make the myelin more vulnerable to injury from Abeta (see below) (Osgood et al., 2017).

### APOE4

The number of oligodendrocytes (OLs) decreases in frontal cortex of APOE4 brains (Cheng et al., 2022). This decrease of OLs was also observed in humanized APOE4 transgenic mice without any neuronal loss. Cultured OLs were killed by lipidated APOE4 (Cheng et al., 2022).

Other evidence for APOE4 causing myelin injury includes the finding of DTI white matter abnormalities in cognitively normal APOE4 homozygotes (Operto et al., 2018) and heterozygotes (Nierenberg et al., 2005). APOE affects microstructural properties of the brain WM from early adulthood (Westlye et al., 2012). In contrast APOE2 elderly cognitively normal carriers have robust white matter by DTI (Chiang et al., 2012). Even one APOE4 allele increases cognitive decline and white matter damage in non-demented elderly (Sun et al., 2020). ApoE4 is associated with atherosclerosis, amyloid angiopathy, and WM damage in AD (Tian et al., 2004) and likely accounts at least in part for the frequent co-occurrence of these conditions in AD patients (Sweeney et al., 2019).

### Abeta

Abeta peptides can kill mature oligodendrocytes (OLs) but not oligodendrocyte precursor cells (OPCs) in culture (Xu et al., 2001; Roth et al., 2005; Horiuchi et al., 2012). Moreover, Abeta peptides also inhibit myelin sheet formation after differentiation of OPCs (Horiuchi et al., 2012). Importantly, mature oligodendrocytes contain APP (Garcia-Ladona et al., 1997) and secrete Abeta1-40 and Abeta 1-42 (Skaper et al., 2009b). The metalloprotease ADAMTS4 found in oligodendrocytes generates N-truncated Aβ peptides and suggests OLs could release amyloidogenic peptides in AD (Zeng et al., 2005; Walter et al., 2019). Abnormalities of this Abeta secretion could lead to white matter injury (Skaper et al., 2009b). Abeta peptides produce OL cell death by activating the sphingomyelinase-ceramide (Lee et al., 2004). One study has suggested Abeta oligomers may remove lipid molecules from the myelin bilayer (Ngo et al., 2021). Indeed, AD senile amyloid plaques have a 1:1 ratio of Abeta and cholesterol (Ngo et al., 2021). Injection of Abeta1-42 into rat brain corpus callosum damages myelin, oligodendrocytes and axons (Jantaratnotai et al., 2003). Soluble Abeta is increased in AD white matter and has no correlation with the number of gray matter amyloid plaques (Collins-Praino et al., 2014). Cerebral amyloid levels are associated with greater white matter hyperintensity progression in cognitively normal older adults (Scott et al., 2016).

It seems possible that Abeta may have a larger role in oligodendrocyte/OPC/myelin injury in some cases of AD. For example, in familial AD (early onset), there are marked increases of brain Abeta that are initially intracellular without extracellular increases that could cause white matter injury prior to any plaque and tangle formation. There are early increases in Abeta in the hippocampus of APP transgenic mice that occur prior to formation of plaques and tangles, which are markedly increased by a high fat and/or high cholesterol diet (Shie et al., 2002, 2003).

### Familial AD genes

Presenilin-1 mutations increase Abeta related oligodendrocyte injury (Desai et al., 2011) and other FAD mutations also appear to have Abeta related WM injury (Zhang et al., 2022; Figure 1). Triple-transgenic AD (3 × Tg-AD) mice, which harbor three FAD mutations, show myelin abnormalities similar to FAD patients, suggesting that Abeta may contribute to white matter injury (Desai et al., 2009). A Presenilin mutation affects OPC differentiation, compromised OPC cell function, affected myelin basic protein distribution, and sensitized the OPCs to Abeta induced injury which was prevented by a GSK-3β inhibitor (Desai et al., 2010, 2011). These results were supported by a previous study showing a Presenilin-1 mutation worsens glutamate and Abeta injury to OLs, worsens WM damage and worsens memory function in mice (Pak et al., 2003). Abeta production is increased with Presenilin redistribution and aberrant cholesterol transport (Burns M. et al., 2003). FAD patients with Presenilin mutations have a number of WM biochemistry abnormalities (Roher et al., 2013).

### Cholesterol

About a quarter of the cholesterol found in the body is in the brain, with most of the brain cholesterol found in myelin. Most of the brain cholesterol is synthesized within the brain itself, with the blood brain barrier isolating peripheral from brain cholesterol. However, there are transporters at the BBB that transport cholesterol from brain to blood and another that transports cholesterol from blood to brain (see below) (Dietschy and Turley, 2001; Björkhem and Meaney, 2004). The brain cell types that synthesize cholesterol shifts from neurons during embryogenesis to oligodendrocytes during postnatal myelination and finally mainly to astrocytes in the adult brain (Saher and Stumpf, 2015).

A study of 403 young normal adults showed that cholesterol levels influence white matter integrity as defined by DTI; moreover, changes of cholesterol- related genes produced age-specific effects in brain (Warstadt et al., 2014). Serum cholesterol can predict DTI white matter microstructure (Warstadt et al., 2014). Patients with familial hypercholesterolemia have a greater incidence of mild cognitive impairment (Zambón et al., 2010) which is associated with white matter abnormalities as assessed by MRI-DTI (Lee et al., 2009; Fischer et al., 2015). Local cholesterol metabolism orchestrates remyelination (Berghoff et al., 2022). Blood cholesterol modestly increases the risk of dementia in a study of more than 1.8 million people over two decades (Iwagami et al., 2021), which could be due in part to its effects on brain white matter. AD patients with the Swedish APP 670/671 mutation have increased 27-hydroxycholesterol in their brains (Shafaati et al., 2011). Since the 27-OH cholesterol ester comes from peripheral blood, this implies peripheral cholesterol influx is increased in brain by this mutation (see below).

### Hypertension and cardiovascular disease

Cardiovascular disease is associated with white matter injury (Suzuki et al., 2021; Austin et al., 2022). Elevated blood pressure even in young adults leads to white matter abnormalities (Maillard et al., 2012). Blood pressure and indicators of brain small-vessel disease are associated with atrophy of structures affected by AD (den Heijer et al., 2005). Hypertension associated with dementia leads to oxidative damage and metabolic dysfunction, systemic inflammation and variability in autonomic control of heart rate (Daugherty, 2021). Spontaneously hypertensive rats have vascular tight junction disruption associated with inflammatory white matter injury (Yang et al., 2018). Age-related differences in cerebral WM are worsened by hypertension (Burgmans et al., 2009). Amyloid angiopathy and hypertension are both associated with white matter lesions by MRI in the aging brain (Scott et al., 2015). Even transient hypertension in midlife can result in white matter lesions and cerebrovascular pathology in rat brain later in life (Lai et al., 2021). Proton magnetic resonance spectroscopy showed similar white matter biochemical changes in patients with chronic hypertension and early Alzheimer’s disease (Catani et al., 2002).

White matter hyperintensity volumes are greater in old adults with low cardiac output due to cardiovascular disease (Jefferson et al., 2007). AD patients have lower cardiac ejection fractions, lower cerebral blood flow and more atherosclerotic plaques in the aorta and brain arteries. Cardiac ejection fraction, basilar artery blood flow velocity and internal carotid artery velocity are associated with AD (Jin W. S. et al., 2017). Low cardiac index is associated with AD (Jefferson et al., 2015). Hypertension, heavy alcohol consumption, and diabetes damage white matter which accelerates cognitive decline in the elderly (Wang et al., 2015).

Experimentally induced brain hypoperfusion induces white matter injury (Farkas et al., 2004; Chen et al., 2022). Bilateral occlusion of the common carotid arteries causes white matter damage in adult rats (Kim et al., 2008). Brain ischemia can be a prelude to AD (Pluta et al., 2021). Vascular dysfunction, in part due to hypertension and atherosclerosis, is an early feature of AD (Sweeney et al., 2019). Of note, there are progressive cerebrovascular abnormalities in an AD mouse model (Jullienne et al., 2022).

### White matter hyperintensities (WMH)

The relationship between WMH and WM injury in AD is not clear. However, WMH lesion volume appears to be a risk factor for developing AD and presumably the WMH injury associated with AD (Defrancesco et al., 2013). White matter hyperintensities predict amyloid increase in Alzheimer’s disease (Grimmer et al., 2012). The spatial distribution of WMH is associated with future amyloid accumulation in the cognitively normal elderly without PET-detectable amyloid pathology (Moscoso et al., 2020). A higher WMH volume is a risk factor for the conversion to AD (Defrancesco et al., 2013). WM disease is one predictor of progression from mild cognitive impairment to AD (Prasad et al., 2011). A meta-analysis of 36 prospective trials showed that WMH increased the risk of developing impaired cognition and frank dementia (Hu et al., 2021).

### Inflammation/Oxidative stress/Inflammasomes

Mature oligodendrocytes (OLs) in both AD patients and an AD mouse model undergo NLRP3-dependent Gasdermin D-associated inflammatory injury to myelin and axons (Zhang et al., 2020). Knockdown of Drp1 (a mitochondrial fission guanosine triphosphatase) in oligodendrocytes (OLs) in AD mice blocks NLRP3 activation, prevents myelin loss, and improves memory function (Zhang et al., 2020). Drp1 activation in OLs decreases glycolysis in AD mouse models by inhibiting hexokinase 1 (HK1), which triggers NLRP3-associated inflammation. Thus, the Drp1-HK1-NLRP3 pathway appears to play a major in OL injury and possibly reducing brain glucose metabolism seen in AD (Zhang et al., 2020).

Serum total antioxidant status assays show increased oxidative stress in AD brain (Zito et al., 2013). The Nrf2 knockout mouse emphasizes the importance of oxidative stress to myelin health. Nrf2 is an oxidant-activated transcription factor that increases the expression of almost every known anti-oxidant gene. Nrf2 knockout mice are normal when young and in midlife develop a diffuse loss of myelin by the time they are 10 months of age (Hubbs et al., 2007). Thus, the survival of myelin into older age is absolutely dependent on the presence of Nrf2 activation of anti-oxidants enzymes, and any decrease of anti-oxidant status associated with aging might account in part for aging related and even AD related myelin injury. The importance of inflammation in AD is emphasized by the fact that Non-Steroidal Anti Inflammatory Drugs (NSAIDS) do not affect the course of AD, but they do markedly decrease the risk of developing AD when taken prior to any clinical symptoms or signs (de Craen et al., 2005; Vlad et al., 2008; Imbimbo, 2009; Nguyen et al., 2022). Given these findings it seems reasonable to suggest that anti-oxidants and NSAIDs might help protect white matter during aging and perhaps delay injury to myelin and delay AD. IL18 has been suggested to be a proinflammatory marker for cerebral white matter injury (Altendahl et al., 2020).

### Diabetes

Diabetes is a surprising but recognized risk factor for AD. APOE4 and diabetes interact to promote injury to later myelinating WM regions in cognitively normal aged adults (Foley et al., 2014). A rodent model of experimental diabetes shows decreased LRP1 (reduced Abeta efflux) and increased RAGE (increased Abeta influx) which led to higher Abeta levels and memory deficits (Ma et al., 2017). Diabetes alters the rat cerebral cortex myelin lipid profile (Cermenati et al., 2017). Diabetes causes microvascular injury which affects white matter (Ly et al., 2017). Dysregulated proteolysis of RAGE and APP in type 2 diabetes mellitus also provides a possible risk factor to AD (Kojro and Postina, 2009). Diabetes mellitus-related behavioral deficits in mice correlate with dysfunction of oligodendrocyte precursor cells (Wang et al., 2022a).

### Lipopolysaccharide (LPS)

We have shown that the combination of LPS with hypoxia followed by brief focal cerebral ischemia in the adult rat resulted in white matter damage in both hemispheres which preceded the formation of amyloid-like plaques in ischemic cortex weeks later (Zhan et al., 2015a). LPS acts at the BBB to increase Abeta influx (not RAGE dependent) and decrease Abeta efflux (LRP1 dependent) (Jaeger et al., 2009), which could participate in amyloid plaque formation (Deane et al., 2009) and contribute to Abeta mediated myelin injury as noted above. Lipopolysaccharide induced sepsis causes amyloid-β plaque formation and tau phosphorylation in cortex and hippocampus of rats (Wang et al., 2018; Kirk et al., 2019).

### LRP1

(low-density lipoprotein receptor-related protein 1)Low-density lipoprotein receptor-related protein 1 is required for survival of oligodendrocyte progenitor cells (OPC) (Schäfer et al., 2019). Since cholesterol can down-regulate LRP1 (Zhou et al., 2021), this could potentially impair function of OPCs and impair myelin repair. OPCs require LRP1 to maintain normal cholesterol and require LRP1 to differentiate into mature oligodendrocytes (OLs) (Lin et al., 2017). OLs and OPCs deficient in LRP1 have increased levels of sterol-regulatory element-binding protein-2 and cannot maintain normal cholesterol levels. Treatment of LRP1 deficient OPCs treated with cholesterol or pioglitazone (to activate PPAR-γ) cannot differentiate. However, treatment with both promotes OPC differentiation into mature OLs (Lin et al., 2017). Thus, changes of LRP1 levels and cholesterol dysmetabolism as shown below in AD could lead to OPC and white matter abnormalities.

### Blood brain barrier/Pericytes

Degeneration of pericytes disrupts blood flow to the white-matter which results in fibrinogen deposition which in turn causes loss of myelin and axons and OLs. This disrupts white matter circuits causing functional loss before loss of neurons (Montagne et al., 2018). Astrocytes not only help maintain the BBB integrity but also synthesize cholesterol, express ApoE, and provide cholesterol to neurons and oligodendrocyte in brain (Saher and Stumpf, 2015; Wang et al., 2018). Thus, astrocyte delivery of cholesterol to oligodendrocytes could affect white matter integrity.

Fibrinogen causes OL cell death in oligodendrocyte and pericyte cultures. Decreasing systemic fibrinogen decreased white-matter fibrinogen deposition, pericyte cell death, vascular injury and white-matter changes. Thus, changes in the BBB and pericytes can result in myelin/WM injury (Montagne et al., 2018). Pericyte deficient mice have increased flux of cholesterol into brain and increased cholesterol synthesis (Saeed et al., 2014) which could impair myelin function. Free cholesterol and APOE cannot pass the BBB, whereas 24-hydrocholesterol can exit brain *via* the BBB and 27-hydroxycholesterol can enter brain *via* the BBB (Rhea and Banks, 2021). APOE deficiency compromises the BBB especially after injury, whereas APOE protects against neuropathology induced by high cholesterol diets and maintains the BBB during aging (Fullerton et al., 2001; Methia et al., 2001; Mulder et al., 2001). Abeta causes BBB dysfunction of vascular endothelial cells which is prevented by LRP6 activation of the Wnt/β-catenin pathways (Wang et al., 2022c).

### Other AD risk factors

A meta-analysis of risk factors that predict progression from mild MCI to AD included: APOE4, CSF tau levels, atrophy of the hippocampus and temporal lobe and entorhinal cortex, diabetes mellitus, high blood pressure, increasing age, traumatic brain injury (TBI), and female sex (Li et al., 2015; Graham et al., 2022; Mielke et al., 2022). Notably, a number of these are also associated with white matter injury. Of interest is the distribution of white matter abnormalities in TBI patients resembles those of with early AD (Fakhran et al., 2013).

### Recent studies linking APOE, cholesterol, and myelination

A recent study by Mok et al. (2022) shows that APOE is localized mainly to astrocytes, and that astrocyte APOE facilitates the transfer of cholesterol to oligodendrocytes which require it for normal differentiation and development. They show that APOE4 decreases the astrocyte transport of cholesterol both *in vivo* and *in vitro* which disrupts oligodendrocyte differentiation (Mok et al., 2022). A study by Wang et al. (2022b) shows that microglia promote myelin debris clearance, but that APOE4 microglia are unable to do this which further disrupts the myelin. Using snRNA-seq on human post-mortem tissue, Blanchard et al. (2022) identified altered lipid homeostasis in APOE4 oligodendrocytes and validated their findings in iPSCs. Thus, APOE4 disrupts normal cholesterol homeostasis in astrocytes and likely neurons which in turn affects oligodendrocyte differentiation and survival and affects developing and mature myelin as well as phagocytosis of damaged myelin.

## White matter injury in ad brain contributes to cholesterol dysmetabolism

Brain contains a fourth of the body’s cholesterol, and cholesterol accounts for ∼1/4 of the lipids found in myelin (Martins et al., 2009; Cantuti-Castelvetri et al., 2018; Sharma et al., 2022). Most cholesterol in brain is synthesized by brain cells – mostly astrocytes in adult brain, with the half-life of the cholesterol being ∼5 years (Martins et al., 2009). Thus, injury to myelin would result in cholesterol dysmetabolism. White matter injury is associated with loss of cholesterol and myelin proteins which is proposed to cause diffuse cholesterol dysmetabolism in myelin in both gray and white matter (Roher et al., 2002). Even plasma lipids, including LDL cholesterol and HDL cholesterol are associated with myelin/white matter injury in AD (Iriondo et al., 2021). Cholesterol is decreased over 70% in AD white matter (Wallin et al., 1989), supporting the idea that myelin injury contributes to cholesterol dysmetabolism (Roher et al., 2002). Notably, defective cholesterol clearance by the transporters discussed below which are down regulated in AD limits remyelination in the aged central nervous system (Cantuti-Castelvetri et al., 2018). Myelin debris clearance requires cholesterol transporters including ApoE. Stimulation of reverse cholesterol transport restores the capacity of old mice to remyelinate lesioned white matter. The cholesterol in myelin debris can overwhelm the ability of phagocytes and microglia to remove the debris. This results in formation of cholesterol crystals and dysfunctional immune response to the debris which impair white matter remyelination (Cantuti-Castelvetri et al., 2018). Thus, cholesterol released from damaged myelin orchestrates remyelination (Berghoff et al., 2022). Myelin-derived lipids including cholesterol act on the liver X receptor (LXR) to regulate macrophage and microglial activity (Figure 2; Bogie et al., 2012).

Though total cholesterol decreases as myelin injury progresses in AD brain, it is likely that the amount of released “free” cholesterol from myelin increases and is immediately bound to APOE which transports cholesterol into cells. Thus, the levels of APOE-bound cholesterol are postulated to be increased in AD brain. This increased cellular cholesterol leads to increased Abeta in endothelial cells, neurons, oligodendrocytes and other cells. This occurs in part because the increased cholesterol in endothelial cells inhibits LRP1 which decreases Abeta egress from brain/endothelial cells to blood, and increased cholesterol activates RAGE which increases Abeta influx from blood to endothelial cells and brain as reviewed in the next section and shown in Figure 2. In addition, as noted above, cholesterol dysmetabolism may contribute to white matter injury itself. If so, this sets a vicious cycle in motion (Figure 1).

## Myelin injury coupled with cholesterol dysmetabolism contribute to Abeta dysmetabolism and ad pathology

### Cholesterol

There is now a considerable literature on how cholesterol and ApoE interact with Abeta synthesis and transport, APP metabolism, amyloid formation and tau phosphorylation (Michikawa, 2006; Carter, 2007; Popp et al., 2013; Allinquant et al., 2014; Gamba et al., 2015; Sun et al., 2015; Fanaee-Danesh et al., 2019; Loera-Valencia et al., 2019; Chew et al., 2020; Chai et al., 2021; Nanjundaiah et al., 2021; Wu et al., 2022). In general higher plasma and brain cholesterol and its metabolites correlate with higher brain Abeta levels and lower CSF Abeta levels (Reed et al., 2014; Iriondo et al., 2020). Increased blood total cholesterol, decreased HDL-C increased LDL-C levels in blood are associated with an increased risk of AD (Tang et al., 2019). Statins decrease the risk of developing AD (Sjögren and Blennow, 2005; Haag et al., 2009; Zhu et al., 2018; Langness et al., 2021) though it is controversial whether statins affect the course of AD (Sjögren and Blennow, 2005; Zhu et al., 2018).

TREM2, a risk factor for AD, affects cholesterol, myelin, and phospholipid metabolism, and promotes the AD microglial phenotype (Li et al., 2022). As many as a third of the genes associated with AD are related to cholesterol metabolism (Carter, 2007; Wollmer, 2010). A risk score based upon the more than 50 AD associated loci associated can predict AD with up to 90% accuracy (Sims et al., 2020) and many of these 50 loci relate to cholesterol metabolism. Transcriptomic data show that the cholesterol gene expression changes found in AD brain are not observed in Parkinson’s disease (PD) brain samples. There are alterations in cholesterol biosynthesis, cholesterol catabolism and cholesterol transport which is accompanied by the accumulation of cytotoxic oxysterols (Varma et al., 2021). APOE4 causes cholesterol dysmetabolism (Jeong et al., 2019).

One human study (Religious Orders Study/Memory and Aging Project) showed that LDL-cholesterol correlated with AD neuropathology and amyloid angiopathy regardless of the of APOE status (Wingo et al., 2022). Finally, CSF 7-Ketocholesterol which is derived from peripheral blood correlates with CSF Abeta levels and DTI white matter abnormalities in cognitively healthy adults (Iriondo et al., 2020).

Lipoprotein receptor 1 (LRP1) regulates brain levels of ApoE and cholesterol (Liu et al., 2007). LRP1 also transports Abeta out of cells and out of the brain (Deane et al., 2009). Deletion of APP or parts of the Ɣ-secretase complex increased LRP1 expression and function. This was blocked by APP intracellular domain (AICD) over expression (Liu et al., 2007). AICD, along with Fe65 and Tip60, bind the LRP1 promoter to decrease it transcription. APP regulates cholesterol and apolipoprotein E metabolism in brain *via* (Liu et al., 2007). Pericytes at the BBB remove aggregated Abeta using a LRP1-dependent APOE isoform specific mechanism (Ma et al., 2018).

Lipopolysaccharide (LPS), found in Gram-negative bacterial cell walls, is increased in AD brain and co-localizes with amyloid plaques and oligodendrocytes (Zhan et al., 2016). LPS also causes cholesterol dysmetabolism by stimulating cholesterol 24-hydroxylase which results in cholesterol efflux from cells and the brain across the BBB, as well as inhibiting 3-hydroxy-3-methulglutaryl-CoA reductase, the rate-limiting enzyme for cholesterol synthesis (Na et al., 2021).

### Cholesterol binding to Abeta and APP fostering formation of amyloid plaques

High cholesterol causes Alzheimer’s amyloid pathology to appear earlier in transgenic AD mouse models (Refolo et al., 2000). Cholesterol and possibly APOE bind Abeta peptide monomers as well as APP (Barrett et al., 2012; Di Scala et al., 2013; Kanekiyo et al., 2014; Panahi et al., 2016; Hashemi et al., 2022). Cholesterol and APOE regulate APP cleavage (Howland et al., 1998; Mills and Reiner, 1999). Mutations in the APP cholesterol-binding site alter APP processing to form less toxic Aβ peptides (Hanbouch et al., 2022). Free cholesterol fosters Aβ self-assembly on membranes (Hashemi et al., 2022), likely along with degraded myelin basic protein as noted below (Zhan et al., 2018). Cholesterol also promotes Aβ42 aggregation through a nucleation pathway (Habchi et al., 2018). Cholesterol oxidation products enhance Abeta aggregation and neurotoxicity (Usui et al., 2009). Cholesterol also promotes Abeta aggregation through formation of an endogenous seed (Yanagisawa, 2003). Cholesterol also interacts with MBP both of which bind APP (Rivas et al., 1998). Cholesterol, APOE and Abeta co-localize in amyloid plaques (Mori et al., 2001; Burns M. P. et al., 2003). Presenilin-1 insufficiency inhibits the normal cleavage of APP (De Strooper et al., 1998). The AD associated C99 APP fragment regulates cholesterol transport (Montesinos et al., 2020).

### Dietary cholesterol and cholesterol transporters

Increased dietary cholesterol intake promotes Abeta formation and AD pathology (Pappolla et al., 2003; Ghribi et al., 2006; Ismail et al., 2017; Liu et al., 2018; Wu et al., 2022) and tau hyperphosphorylation (Bhat and Thirumangalakudi, 2013; Park et al., 2013) and cognitive impairment (Umeda et al., 2012). Decreased cholesterol biosynthesis decreases γ-secretase activity and decreases Aβ generation (Kim et al., 2016). Hypercholesterolemia increases Abeta production by increasing BACE1 and RAGE levels, and decreasing IDE (Insulin Degrading Enzyme) and LRP1 levels (Jaya Prasanthi et al., 2008). Peripheral cholesterol metabolism is generally quite independent of central cholesterol metabolism, with the exception that 27-hydroxycholesterol can enter brain from blood (Mahalakshmi et al., 2021), presumably accounting for dietary effects of cholesterol on Abeta metabolism and cognition (Heverin et al., 2015; Zhang et al., 2018). Increased 27-hydroxycholesterol uptake by brain causes decreased glucose uptake, perhaps contributing to the glucose hypometabolism associated with AD (Gamba et al., 2019). In addition, 24-hydroxycholesterol can exit brain accounting for decreased Abeta and amyloid plaques (Brown et al., 2004). Indeed, 24-hydroxycholesterol levels in CSF are increased in AD compared to controls (Schönknecht et al., 2002).

The three main cholesterol exporters from brain are cholesterol 24-hydroxylase from the CYP46A1 gene, ABCA1 and ABCG1 (Panzenboeck et al., 2002; Brown et al., 2004; Ohtsuki et al., 2007; Figure 2). ABCA1- and ABCG1-mediated cholesterol transport out of brain into the CSF is impaired in AD (−73 and −33%, respectively) which would tend to increase brain cholesterol which in turn would affect Abeta/amyloid metabolism (Marchi et al., 2019; Figure 2). In addition, a polymorphism of the CYP46 cholesterol export gene increased Abeta and Tau and increased the risk of AD (Papassotiropoulos et al., 2003). Increasing APOE levels and ABCA1, its lipid transporter, increase the clearance of Aβ from brain (Wildsmith et al., 2013). High cholesterol diets produce BBB dysfunction (Takechi et al., 2013) which would tend to decrease clearance of both Abeta and cholesterol from brain.

27-hydroxycholesterol which is derived from the blood and APOE4 activate the C/EBPβ/δ-secretase pathway to form amyloid plaques (Wang Z. H. et al., 2021). 27-hydroxycholesterol promotes Aβ accumulation in mild cognitive impairment patients and in the APP/PS1 mouse AD model (Zhang et al., 2019). Increasing blood 27-Hydroxycholesterol modulates brain cholesterol metabolism and impairs learning and memory in rats (Zhang et al., 2015) and mice (Heverin et al., 2015). Since cholesterol cannot cross the BBB while 27-hyroxycholesterol does cross, it is likely that hypercholesterolemia increase in AD risk is accounted for by influx of 27-hydroxycholesterol from blood to brain (Heverin et al., 2005, 2015; Björkhem et al., 2006; Shafaati et al., 2011; Zhang et al., 2015, 2018, 2019; Gamba et al., 2019; Wang Z. H. et al., 2021; Wang et al., 2022d; Wu et al., 2022).

A short hairpin RNA directed against Cyp46a1 mRNA using an AAV vector decreased expression of the Cyp46a1 gene in neurons of normal mice and increased cholesterol in the neurons (Djelti et al., 2015). This produced apoptotic cell death, hippocampal atrophy and memory impairments which were associated with APP recruitment to lipid rafts which increased Abeta and Tau (Djelti et al., 2015). The same group found that Abeta increased in the brain of the APP23 mouse AD model of AD following inhibition of Cyp46a1 expression, one of the cholesterol transporters (Figure 2; Djelti et al., 2015).

### Cholesterol and tau

An analysis of protein co-expression from Tau transgenic mice and AD brains identified four highly associated modules including cholesterol biosynthesis (Tsumagari et al., 2022). Pathogenic tau mutations upregulate cholesterol synthesis pathways (Glasauer et al., 2022). DHCR24, which is synthetase 3β-hydroxysterol-Δ24 reductase (DHCR24), regulates cholesterol synthesis and metabolism. DHCR24 knockdown activates Ras/MEK/ERK signaling which causes tau hyperphosphorylation (Mai et al., 2022). Dietary cholesterol induces higher levels of tau and tau hyperphosphorylation (Wang et al., 2022d). The levels of CYP46A1 and 24S-hydroxycholesterol in the hippocampus are lower in the THY-Tau22 mouse AD model which would explain the higher brain cholesterol in these mice (Burlot et al., 2015). Increasing the CYP46A1 and 24S-hydroxycholesterol levels with AAV vectors improve the cognitive deficits and long-term depression in the THY-Tau22 mouse AD model (Burlot et al., 2015). A high cholesterol diet induces tau hyperphosphorylation in APOE deficient mice (Rahman et al., 2005). P-tau181 levels independently predict the CSF desmosterol, cholesterol and 24S-hydroxycholesterol concentrations in AD patients (Popp et al., 2013).

### APOE/Cholesterol effects on Abeta/APP

Various studies suggest APOE genotype affects Abeta clearance and deposition by direct binding with APP. However, at least one study suggests ApoE affects amyloid-β (Aβ) export in spite of little evidence of direct APOE and Aβ association in their experimental paradigm (Verghese et al., 2013). However, two apolipoprotein E mimetic peptides have been shown to directly bind LRP1 and presumably regulate its ability to transport Abeta out of the brain (Croy et al., 2004). Another study appeared to show direct binding of intact APOE to LRP1 (Zhu and Hui, 2003). A novel APOE blocked the interaction of APOE and the N-terminal of APP, reduced Abeta pathology and improved memory functions in an AD mouse model (Sawmiller et al., 2019).

Cholestenoic acid, a cholesterol metabolite, decreases γ-secretase activity (Jung et al., 2015). Changes in membrane cholesterol decrease γ-secretase activity and Aβ (Kim et al., 2016). Inhibition of ACAT (a family of enzymes that converts membrane cholesterol into esters for cholesterol storage and transport) decrease brain Aβ (Puglielli et al., 2001, 2004; Bhattacharyya and Kovacs, 2010; Bryleva et al., 2010). An ACAT inhibitor decreases amyloid plaques in a mouse AD model (Hutter-Paier et al., 2004). Ablating the ACAT1 gene increases 24 (S)-hydroxycholesterol content (which should decrease brain cholesterol) and decreases amyloid plaques in a mouse AD model (Bryleva et al., 2010). Peripheral liver APOE4 can exert adverse effects on the normal and AD brain independent of the brain allele (Liu C. C. et al., 2022).

### Cholesterol effects on Abeta transporters

Low-density lipoprotein receptor-related protein 1 is the main transporter for Abeta out of cells and out of brain *via* the BBB (Shibata et al., 2000; Zlokovic et al., 2010). Hypercholesterolemia decreased LRP1 expression, which would decrease Abeta efflux across the BBB, and increased RAGE expression, which would increase Abeta influx through the BBB, in cerebral endothelial cells (Zhou et al., 2021). Hypercholesterolemia increased brain apoptosis in AD mice. In an *in vitro* experiment, increasing cholesterol decreased LRP1, increased RAGE, and increased Abeta in cerebral endothelial cells. These effects were mediated by Wnt/β-catenin signaling pathway acting on the LRP1 and RAGE promoters (Zhou et al., 2021). Other studies have also shown that cholesterol, which is transported by APOE, decreases LRP1 and increases RAGE to increase Abeta in endothelial cells, neurons and glia (Cutler et al., 2004; Mosconi et al., 2008). Cholesterol regulates metalloproteinase mediated shedding of LRP1 (Selvais et al., 2011). APOE4 is not as effective as other ApoE isoforms in regulating LRP1 shedding, which may help explain the different abilities of these isoforms to remove Aβ from brain (Bachmeier et al., 2014). LRP1 and APOE mRNA levels are elevated in AD brain (Akram et al., 2012), perhaps in response to increased levels of Abeta (LRP1 transporter) and Cholesterol in AD brain. LRP1 modulates Wnt signaling to affect cholesterol storage and fatty acid synthesis (Terrand et al., 2009). Notably, LRP1 controls phosphorylation of cPLA2 which in turn modulates ABCA1 expression and cholesterol export from cells and from the brain (Zhou et al., 2009). This shows the intricate relationship between Abeta and cholesterol transport and means that changes in one will affect transport of the other (Loera-Valencia et al., 2019; Figures 1, 2).

Astrocyte-Derived Cholesterol Regulates Abeta Production in Neurons. Astrocyte-derived cholesterol together with APOE facilitates the movement of neuronal APP in and out of lipid rafts to interact with beta and gamma secretases to form Abeta (Wang H. et al., 2021). Preventing cholesterol synthesis by astrocytes decreases amyloid plaques and phosphorylated tau in an AD mouse model (Wang H. et al., 2021). Treating astrocytes with cholesterol-free APOE or decreasing cholesterol synthesis in cultured neurons causes APP to migrate out of lipid clusters allowing it to interact with alpha secretase which produces soluble APP, which protects neurons against Abeta injury. Thus, astrocyte regulation of cholesterol metabolism produces high cholesterol levels in astrocytes but low cholesterol levels in neurons which inhibits Abeta formation in neurons (Wang H. et al., 2021). Thus, astrocytes play a central role in regulating cholesterol metabolism in the adult brain, and thereby modulates amyloid metabolism to presumably protect neurons and possibly oligodendrocytes against Abeta toxicity (Staurenghi et al., 2021).

### ABCA and other ABC transporters (cholesterol and Abeta)

As noted above, LRP1 modulates cPLA2 phosphorylation, ABCA1 expression and export of cholesterol out of cells (Zhou et al., 2009). Downregulation of ABCA7 modifies cholesterol metabolism and decreases Aβ peptide efflux and promotes amyloid plaque formation in an *in vitro* BBB model (Lamartinière et al., 2018). ABCA1- and ABCG1-mediated efflux of cholesterol from brain to CSF is decreased in AD (Marchi et al., 2019). Lipidation of ApoE by ABCA1 is needed for the RXR agonist bexarotene to clear Abeta and improve memory deficits (Corona et al., 2016). A mutation in ABCA1 found in 1 in 500 subjects is associated with low APOE plasma levels and a high AD risk and high risk of cerebrovascular disease (Nordestgaard et al., 2015). Brain pericytes ABCA1 exports cholesterol but has no effect on Abeta (Saint-Pol et al., 2012). ABCG1 and ABCA1 regulate efflux of cholesterol from neurons to APOE and decrease formation of amyloid plaques (Kim et al., 2007; Behl et al., 2021). Of note, one study found that Tangier disease ABCA1 mutants modulate cellular amyloid-β production independent of any effect on cholesterol (Kim et al., 2011). ABCA1 deficiency decreases brain ApoE and increases amyloid plaque formation in APP23 mice (Koldamova et al., 2005). ABCA1 binds APOE and then increases cholesterol transport across the BBB. Decreased ABCA1 function increases Abeta deposition and increased ABCA1 decreases formation of amyloid plaques (Wollmer et al., 2003; Martins et al., 2009). Abcg4 at the mouse BBB decreases Abeta entry into brain, a process antagonized by cholesterol (Dodacki et al., 2017). ABCB1 and ABCA1 increase Abeta export from brain, which is also antagonized by cholesterol (Elali and Rivest, 2013). ABCA1 and ABCG1 export cholesterol from astrocytes but not from neurons and ABCG4 exports cholesterol from neurons but not astrocytes (Chen et al., 2013). ABCA1, along with cholesterol 24-hydroxylase/CYP46A1, are mainly responsible for cholesterol efflux from brain to blood at the BBB (Do et al., 2011; Saint-Pol et al., 2012). Decreasing the function of either increases brain cholesterol which increases Abeta by decreasing LRP1 and increasing RAGE which promotes amyloid plaque formation (Figure 2).

Cellular Localization of the Molecules in the Model (Figure 2). Though there has not been a systematic study of the cellular localization of the molecules listed in Figure 2 in human AD brain, more evidence is coming to light. For example, APOE appears to be mainly associated with astrocytes (Mok et al., 2022) and cholesterol synthetic genes are localized mainly to astrocytes (Glasauer et al., 2022). LXR, ABC and BACE are expressed in neurons, glia and endothelial cells (Chen et al., 2013). Oligodendrocytes along with neurons and astrocytes synthesize APP and Abeta (Skaper et al., 2009a). Cholesterol derived from astrocytes regulates Abeta production in neurons (Wang H. et al., 2021). LRP1 and RAGE are expressed in most cells in brain (Gaultier et al., 2009), but play a key role in endothelial cells where they regulate the ingress and egress of Abeta to brain *via* the BBB. Pericytes remove Abeta *via* a LRP1-APOE isoform specific mechanism (Ma et al., 2018). Microglia phagocytose APP *via* the LPS CD14 receptor (Liu et al., 2005).

As mentioned, ABCA1, along with cholesterol 24-hydroxylase/CYP46A1 and ABCG1, are mainly responsible for cholesterol efflux from brain to blood at the BBB (Do et al., 2011; Saint-Pol et al., 2012; Marchi et al., 2019), with ABCA1 localized in pericytes (Saint-Pol et al., 2012). In addition, cholesterol export in astrocytes is induced by lipid-free apolipoproteins and lipoproteins, while cholesterol export from neurons occurs only by lipoproteins (Chen et al., 2013; Jeong et al., 2019). ABCA1 and ABCG1 regulate cholesterol export from astrocytes but not neurons (Chen et al., 2013; Jeong et al., 2019; Sierri et al., 2021). ABCG4, which is highest in neurons, regulates cholesterol export only from neurons (Chen et al., 2013; Jeong et al., 2019). Microglia play a role in regulating cholesterol metabolism through the TREM2 receptor (Li et al., 2022).

In normal brain immunocytochemical studies show cholesterol hydrolases CYP46A1 and CYP27A1 in neurons and some astrocytes, and CYP27A1 in oligodendrocytes (Brown et al., 2004). In contrast, in AD brain CYP46A1 is in astrocytes and around amyloid plaques, whereas CYP27A1 decreased in neurons, increased in oligodendrocytes, and was present around amyloid plaques (Brown et al., 2004).

## White matter injury and myelin basic protein in AD

Myelin basic protein Affects Abeta/APP metabolism, and Degraded MBP aggregates and binds Abeta to form plaques. There are decreased levels of soluble APP (sAPPα) in brains of Shiverer (shi/shi) MBP deficient mice, though total APP and sAPPβ were unchanged (Seiwa et al., 2021). The reduced sAPPα was likely due to disintegrin and metalloproteinase-9 (ADAM9) catalysis and non-amyloidogenic processing of APP. MBP -/- mice have increased production of Abeta (Seiwa et al., 2021). However, the MBP-/- mice have virtually no amyloid plaques which we propose is due to the fact that plaque formation may require aggregation of MBP and cholesterol with Abeta (see next section).

### Myelin basic protein

Myelin basic protein (MBP) is an integral part of myelin. In an early study MBP in AD brain was associated with neuronal fractions and neurofilaments (Selkoe et al., 1981). MBP binds Abeta and APP (Hoos et al., 2009; Kotarba et al., 2013) and intact MBP can degrade Abeta (Liao et al., 2009; Mitew et al., 2010; Ou-Yang et al., 2015). LRP1 at the BBB removes degraded MBP (dMBP) from the CNS (Gaultier et al., 2009). In our LPS-ischemia-hypoxia rat AD model we found dMBP surrounding vessels weeks before the formation of amyloid-like plaques (Zhan et al., 2015a). Thus, white matter (WM) injury would produce higher levels of dMBP which would compete with Abeta for removal from the CNS, and thus tend to elevate Abeta levels. That is, WM injury would elevate brain Abeta. It is conceivable that other molecules from damaged myelin (e.g., PLP, MOG, and MAG) might also bind LRP1 to compete with Abeta and elevate Abeta levels in brain.

In addition, we discovered in our LPS-hypoxia-ischemia rat model that dMBP occurred prior to the appearance of amyloid-like plaques (Zhan et al., 2015a). Once amyloid-like plaques formed in this model, they co-localized with dMBP. We speculated that dMBP formed aggregates and helped aggregate Abeta into plaques (Zhan et al., 2015a). This is consistent with studies of multiple sclerosis which have shown that MBP forms aggregates in areas of demyelination (Frid et al., 2015).

It is notable that intact MBP, the N-terminal region, reduces fibrillar amyloid-beta deposition in the Tg 5xFAD mouse model (Ou-Yang et al., 2015) by direct binding to Abeta (Hoos et al., 2009; Kotarba et al., 2013). In addition, the N-terminal regions of MBP can prevent beta amyloid fibrillar assembly and degrade Abeta (Liao et al., 2009; Ou-Yang et al., 2015). This likely accounts for the fact that most amyloid plaques are not found in intact myelin and in fact are found in poorly myelinated regions and demyelinated regions of gray matter (Mitew et al., 2010; Schmued et al., 2013). This also accounts for the fact that there is no intact myelin around plaques in LOAD, early onset familial AD, and in mouse AD models (Mitew et al., 2010). That is, intact MBP in intact myelin would degrade Abeta so that amyloid plaques could not form; plaques can only form in demyelinated regions of gray or white matter (Liao et al., 2009; Mitew et al., 2010; Ou-Yang et al., 2015). This also likely accounts for the fact that amyloid plaques in rodent and human brains are associated with degraded MBP and probably not intact full length MBP (Zhan et al., 2014, 2015a,b). Importantly, the absence of MBP almost completely eliminated the formation of amyloid plaques (Ou-Yang and Van Nostrand, 2013), a finding we interpret to mean that dMBP was not present to help aggregate Abeta into plaques. As noted above, cholesterol plays role in aggregating Abeta as well as MBP (Banerjee et al., 2021; Hashemi et al., 2022).

Of note, antibodies to MBP in AD are 11 times more abundant than controls and found in 16 of 18 AD cases compared to 7 of 90 controls (Singh et al., 1992). This implies there is a net efflux of MBP from brain to blood in AD, and this efflux is through the LRP1 receptor as noted above, which would compete with Abeta and elevate brain Abeta.

## Reversing white matter injury improves cognition in animal AD models

LINGO-1 negatively regulates oligodendrocyte differentiation and myelination and is increased in AD brain. Using the APP/PS1 mouse AD model, an anti-LINGO-1 antibody was shown to improve memory function which was associated with fewer LINGO-1 cells and amyloid plaques but with increased numbers of OPCs and oligodendrocytes and increased myelin density (Yang et al., 2022). A prior study showed no Aβ deposition in 1-month-old 5XFAD mice, but they did have spatial memory deficits associated with demyelination in limbic structures. The same LINGO-1 antibody decreased the myelin injury and improved memory deficits (Wu et al., 2018). A flavenol antioxidant improved memory in 3 × Tg-AD mice, which correlated with fewer amyloid plaques, increased myelin-related gene expression and decreased myelin damage (Yu et al., 2022).

Another recent study showed the rate of new myelin formation was markedly increased in APP/PS1 mice (Chen et al., 2021). Despite this increase, overall myelination levels were decreased in brains of APP/PS1 mice and human AD brains (Chen et al., 2021). To combat this, myelin renewal was enhanced by deleting the muscarinic M1 receptor in oligodendroglia or by giving animals the pro-myelinating drug clemastine. Both treatments markedly improved memory tasks in APP/PS1 mice and increased hippocampal sharp waves. The improved memory function occurred even though the numbers of amyloid plaques and microglia were unaffected by the treatments (Chen et al., 2021). Taken together, these results demonstrate the potential of enhancing myelination as a therapeutic strategy to improve AD-related memory deficits.

Exercise affects myelin in mouse AD models as well. Running decreases the loss of myelinated fibers in hippocampus in the APP/PS1 mouse AD model (Chao et al., 2015). Exercise prior to the onset of AD pathology prevents the memory loss and loss of myelin in white matter in the APP/PS1 mouse AD model (Zhang et al., 2017). Physical exercise may improve cognitive function slightly in AD patients (Liu W. et al., 2022).

## Questions and future studies

The current data suggest that elevated brain cholesterol produced in part by myelin injury appears to be bad for the AD brain. This occurs in part because elevated cholesterol acts to increase Abeta in brain. However, the roles of individual cells from the astrocytes that synthesize most of the cholesterol in adult brain to the endothelial cells that regulate cholesterol and Abeta influx and efflux need to be better understood. How do microglia which phagocytose damaged myelin deal with the cholesterol, and how do cholesterol and oligodendrocytes interact. Does elevated cholesterol accelerate and worsen AD pathology in FAD, LOAD and mouse AD models. Can preventing myelin injury prevent AD. Experiments addressing these and many other questions raised by the model in Figures 1, 2 are sure to extend our knowledge and hopefully help lead to approaches to ameliorate, cure or better yet prevent FAD and LOAD.

## Conclusion

In addition to Abeta dysmetabolism, there is cholesterol dysmetabolism and white matter injury in AD. Moreover, underlying genetics including FAD genes, APOE4 and AD risk factor genes play critical roles in determining whether white matter injury or cholesterol dysmetabolism or Abeta dysmetabolism lead to AD neuropathology and dementia. This review makes the new connection that white matter injury contributes to cholesterol dysmetabolism and that both can drive AD neuropathology with the appropriate genetic predisposition. Finally, Abeta dysmetabolism can also contribute to white matter injury resulting in a vicious injury cycle that may be difficult to slow, halt or reverse. Importantly, cholesterol metabolism and white matter injury provide alternative treatment and prevention targets in AD.

## Data availability statement

The original contributions presented in this study are included in the article/supplementary material, further inquiries can be directed to the corresponding author.

## Ethics statement

The studies involving human participants were reviewed and approved by the University of California at Davis. The patients/participants provided their written informed consent to participate in this study.

## Author contributions

FS conceived of the hypothesis and wrote the first draft of the manuscript. CD, L-WJ, and XZ provided comments and suggestions for improvement. All authors agreed to the final version of the manuscript and agreed to be held accountable for every aspect of the work by ensuring that issues related to the accuracy and integrity of any portion of the manuscript were investigated and resolved.
